# A new type of power energy for accelerating chemical reactions: the nature of a microwave-driving force for accelerating chemical reactions

**DOI:** 10.1038/srep25149

**Published:** 2016-04-27

**Authors:** Jicheng Zhou, Wentao Xu, Zhimin You, Zhe Wang, Yushang Luo, Lingfei Gao, Cheng Yin, Renjie Peng, Lixin Lan

**Affiliations:** 1 Key Laboratory of Green Catalysis and Chemical Reaction Engineering of Hunan Province, School of Chemical Engineering, Xiangtan University, Xiangtan 411105, Hunan Province, China

## Abstract

The use of microwave (MW) irradiation to increase the rate of chemical reactions has attracted much attention recently in nearly all fields of chemistry due to substantial enhancements in reaction rates. However, the intrinsic nature of the effects of MW irradiation on chemical reactions remains unclear. Herein, the highly effective conversion of NO and decomposition of H_2_S via MW catalysis were investigated. The temperature was decreased by several hundred degrees centigrade. Moreover, the apparent activation energy (Ea’) decreased substantially under MW irradiation. Importantly, for the first time, a model of the interactions between microwave electromagnetic waves and molecules is proposed to elucidate the intrinsic reason for the reduction in the Ea’ under MW irradiation, and a formula for the quantitative estimation of the decrease in the Ea’ was determined. MW irradiation energy was partially transformed to reduce the Ea’, and MW irradiation is a new type of power energy for speeding up chemical reactions. The effect of MW irradiation on chemical reactions was determined. Our findings challenge both the classical view of MW irradiation as only a heating method and the controversial MW non-thermal effect and open a promising avenue for the development of novel MW catalytic reaction technology.

What happens when MW irradiation is used for chemical reactions? Since the use of MWs for synthesis reactions first appeared in 1986^1^, numerous studies have investigated the use of MW heating or MW irradiation for organic and materials synthesis, and significant improvements that cannot be obtained by conventional heating methods have been reported^2^,^3^,^4^,^5^,^6^,^7^,^8^,^9^,^10^,^11^,^12^,^13^,^14^. In addition, MW irradiation has been successfully applied in a range of heterogeneous catalytic reaction systems and other reaction systems^15^,^16^,^17^,^18^,^19^,^20^,^21^,^22^,^23^,^24^,^25^,^26^,^27^,^28^,^29^,^30^. Although the use of microwave irradiation to enhance chemical reactions is growing at a rapid rate, the intrinsic nature of the effect of microwave irradiation on chemical reactions remains unclear.

Recently, much interest has been focused on understanding the nature/role of MW reaction rate enhancements^4^,^5^,^8^,^13^,^31^. However, the intrinsic reasons for the MW reaction rate enhancements are still speculative, often conflicting^4^,^5^,^8^,^13^. In particular, there is ongoing debate in the scientific community regarding whether the observed enhancements are the result of purely thermal effects arising from rapid heating and high bulk reaction temperatures generated in the reaction medium due to the radiation field or whether some effects are connected to so-called specific or non-thermal microwave effects, which are related to interactions between the microwave electromagnetic field and the reactant molecules^4^,^5^,^8^,^13^. Few studies have focused on decreasing the Ea’ under microwave irradiation^32^,^33^,^34^, which illustrates a significant microwave catalytic effect. Furthermore, MW irradiation exhibits a MW selective effect^28^,^29^,^30^,^35^.

Previously, we reported that the decrease in the Ea’ rather than solely the “hot-spots” hypothesis is a new reason for microwave-accelerated heterogeneous gas-phase catalytic reactions^36^. However, to the best of our knowledge, no studies on the intrinsic reason for the decrease in the Ea’ under microwave irradiation have been reported. Therefore, the resolution of this important and fundamental question in the field of MW chemistry remains a significant challenge.

Herein, we report the highly effective conversion of NO and decomposition of H_2_S via MW catalysis as case studies. Moreover, a model of the interactions between microwave electromagnetic waves and molecules has been proposed to elucidate the intrinsic reason for the decrease in the Ea’ under microwave irradiation, and a formula for the quantitative estimation of the decrease in the Ea’ was determined. More importantly, this model can be applied to all fields of microwave chemistry. Our findings significantly reveal that MW irradiation can increase the rate of chemical reactions by direct reduction of the Ea’ that is required to activate reactant molecules, and MW irradiation is a new type of power energy for speeding up chemical reactions. Our findings significantly reveal the nature of the interactions between MW irradiation and molecules in chemical reaction systems.

A series of microwave direct catalytic decomposition of NO experiments using CuO-Cu-ZSM-5 as the MW catalysts were conducted (Tables 1 and 2). The highest NO conversion was 65% for the Cu-ZSM-5 catalyst at a residence time (τ) of 2.25 s and a bed temperature of 550 °C in the conventional reaction mode (CRM). However, the highest NO conversion was 98% for the CuO-Cu-ZSM-5 MW catalyst at τ = 2.25 s and a bed temperature of 300–350 °C in the microwave catalytic reaction mode (MCRM). The difference in the catalytic reaction temperature was ~200 °C. We also investigated NO decomposition over the BaMnO_3_ MW catalyst. The highest NO conversion was 93.7% for the BaMnO_3_ catalyst at 250 °C in the MCRM, and the highest NO conversion was only 45.4% at 650 °C in the CRM (Table 1). The difference in the reaction temperature for the NO reduction with (activated carbon) AC in the CRM and MCRM is shown in Table 1.

Similarly, our results indicate that the difference in the catalytic reaction temperature was 200 °C for H_2_S decomposition over the CoS/γ-Al_2_O_3_/BaMn_0.2_Cu_0.8_O_3_ catalyst at Y_H2_ of 6% (see Fig. S5). Zhang^14^,^15^ also reported that the difference in the catalytic reaction temperature was ~200 °C for H_2_S decomposition over the MoS_2_/γ-Al_2_O_3_ catalyst, and the difference in the catalytic reaction temperature was ~200 °C for the catalytic reduction of SO_2_ with CH_4_ over the MoS_2_/Al_2_O_3_ catalyst^14^. Moreover, the difference in the catalytic reaction temperature for other reactions has also been reported^19^,^20^,^22^,^23^.

Surprisingly, Cu-ZSM-5, ZSM-5 and CuO accelerate the decomposition of NO under MW irradiation (See Fig. S3). The Cu-ZSM-5 catalyst exhibits catalytic activity for the decomposition of NO but ZSM-5 and CuO have almost no activity in the CRM. The results indicate that MW irradiation exhibited catalytic effects rather than the catalysis of the catalysts themselves. However, Cu-ZSM-5 exhibited a higher catalytic activity for the decomposition of NO, its catalytic performance could be tuned through the addition of CuO to adjust its MW absorbing property. The MW absorbing property of the CuO-Cu-ZSM-5 catalyst is stronger than that of the single Cu-ZSM-5 catalyst (Fig. S5), and the corresponding catalytic performance of CuO-Cu-ZSM-5 is much higher than that of Cu-ZSM-5. MW irradiation contribute to the catalytic effect, and the different MW absorbing properties of catalysts with the same catalytically active component (Cu-ZSM-5) leads to differences in their catalytic performance under MW irradiation (See Figs S2a,S4). Similarly, the different MW absorbing properties of BaMnO_3_ and BaFeO_3_ resulted in different catalytic performances in the MCRM even though in the CRM, these two catalysts exhibit extremely similar catalytic activities (See Fig. S4). The different MW absorbing properties of the catalysts with the same catalytically active component result in a different catalytic performance under MW irradiation.

At the same time, a certain MW power (P_MW_) corresponds to a balanced catalyst bed temperature under MW irradiation (see Tables S3, S4, S5 and S6).

These results indicated that the reaction temperature decreased by as much as hundreds of degrees centigrade under MW irradiation. The energy comes only from the MW irradiation. To confirm the energy difference in hundreds of centigrade temperature due to MW irradiation compared to the conventional heating mode that results in a decrease in the Ea’, we calculated the Ea’ for these catalysts. Ea’ was 20–25 kJ/mol for CuO-Cu-ZSM-5 (Table 2) in the MCRM, and Ea’ was 71–123 kJ/mol for Cu-ZSM-5 in the CRM. Furthermore, we also determined that the Ea’ decreased from 194.5 kJ/mol in the MCRM to 33.4 kJ/mol in the CRM over BaMnO_3_ (Table 3), and the Ea’ for NO reduction by activated carbon (AC) and H_2_S decomposition (Tables S1 and S2) decreased under MW irradiation. The Ea’ decreased under MW irradiation, indicating that MW irradiation exhibited a significant MW catalytic effect. A portion of the MW energy contributes to the decrease in the Ea’ of reactions under MW irradiation. Therefore, MW irradiation is a new type of power energy for speeding up chemical reactions via the direct reduction of the Ea’ (Fig. 1).

Similarly, few studies have reported a decrease in the Ea’ in other reaction systems under microwave irradiation. Kong *et al.*^32^ reported a decrease in the apparent activation energy for the C + NO reaction (non-catalytic reaction system), which may be due the catalytic effects of MW irradiation. Furthermore, some studies only calculated the apparent activation energy under MW irradiation. Stiegman *et al.*^37^ reported that the apparent activation energy decreased from 118.4 kJ/mol under conventional convective heating to 38.5 kJ/mol under MW irradiation for the carbon-carbon dioxide (Boudouard) reaction (non-catalytic reaction system). Falamaki *et al.*^33^ studied the MW synthesis of colloidal silica using a sodium silicate silica source. In this case, the apparent activation energy was 2.92 kcal/mol under MW irradiation, which was significantly lower than that for conventional synthesis (>10 kcal/mol). However, Stiegman *et al.*^37^ and Falamaki *et al.*^33^ did not provide an explanation for the decrease in the apparent activation energy under MW irradiation. In addition, some studies have predicted a decrease in the activation energy in catalytic reaction systems under MW irradiation including References 38,39.

Based on these results, it is found that MW irradiation is a new type of power energy for speeding up chemical reactions. This new approach is due to MW irradiation directly decreasing the Ea’, which differs from the two typical approaches for speeding up chemical reactions (catalysts and heating transfer energy). In fact, MW irradiation, which results from polarization (polarization of dipole and polarization of interface), is different from conventional heating, which leads to a change of the internal energy level of molecules to reach the activated state. Moreover, a certain MW power (P_MW_) corresponds to a balanced catalyst bed temperature under MW irradiation (see Tables S3, S4, S5 and S6). If all of the MW energy was released by the MW thermal effect, the temperature of the catalyst bed would continue to increase, and there is inexistence of a corresponding balanced catalyst bed temperature. Furthermore, a decrease of several hundreds of degrees centigrade in the reaction temperature under microwave irradiation was observed, and the Ea’ decreased substantially under MW irradiation. These results indicate that a portion of the MW energy was adsorbed by the molecules and another portion of the MW energy was released by the MW thermal effect.

How does MW irradiation directly reduce the Ea’ to speed up chemical reactions? To elucidate the nature of this new type of microwave-driving force for speeding up chemical reactions, we propose a model of the interactions between MW electromagnetic waves and molecules. The Ea_MW_ (reduced directly by MW irradiation) is expressed in equation (9) based on this model. Ea_MW_ is a portion of the MW energy power (P_MW_) that directly interacts with chemical or catalytic reaction systems to reduce the Ea’ under MW irradiation.

This model has four hypotheses as follows: (1) Matter is ideal and uniform, molecular matter is defined as the action mass point, and each action mass point only acts with each “energy molecular”. (2) One portion of the MW energy was adsorbed by the molecules. (3) Another one portion of the MW energy was released by the MW thermal effect to the chemical reaction systems. (4) Under certain conditions, there is a dynamic balance between the adsorbed and released MW “energy molecular” on action mass points.

When a chemical reaction system is irradiated by MWs, “Energy molecular” will interact with the action mass points. Moreover, the adsorbed and released MW “energy molecular” on action mass points will occur simultaneously. Therefore, one portion of the MW energy directly interacts with the molecular matter to reduce the Ea’ for chemical or catalytic reactions. In addition, another portion of the MW energy interacts with action mass points to increase the temperature of the reaction system or catalyst bed, leading to release of this portion of the MW energy by the MW thermal effect.

Each “energy molecular” interacts with each action mass point, which is associated with the MW electromagnetic field (E_MW_) and P_MW_, which can be expressed as k_1_ E_MW_P_MW_:


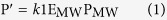


*θ* is the fraction of action mass points occupied by “energy moleculars”, which can be expressed as follows:


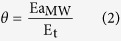


E_t_ is the total energy when all of the action mass points are occupied by “energy moleculars” (i.e., total energy adsorbed by the matter under MW irradiation).

Then, how much “energy molecular” was adsorbed? The rate of “energy molecular” adsorption is proportional to the adsorption of “energy molecular” (*P*′) and the free action mass point (1 − *θ*), which can be expressed as follows:


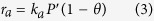


How much “energy molecular” was released? The rate of “energy molecular” release is proportional to action mass points occupied by “energy molecules” (*θ*), which can be expressed as follows:


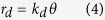


Under MW irradiation, under certain P_MW_ conditions, a dynamic balance exists between the adsorption rate and release rate of MW “energy molecular” on the action mass point in the chemical reaction systems or catalysts. Therefore, the rate of “energy molecular” adsorption is equal to the rate of “energy molecular” release.


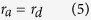



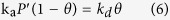



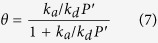



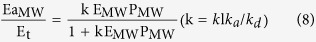



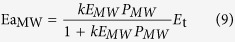


Ea_MW_ can be calculated using Eq. (9). The decrease in the Ea’ can be calculated using Eq. (10).


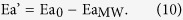


Ea_0_ is the activation energy barrier of the chemical reactions without MW irradiation. K is determined by the dielectric properties of the matter or MW catalysts (Table 3 and S section I) and the MW frequency.

To understand this new type of power energy, the ability of MW irradiation to accelerate chemical reactions via direct reduction of the Ea’ is explained below. Upon MW irradiation of a chemical reaction, the reaction system or MW catalyst adsorbs MW energy and interacts with the MWs. A portion of P_MW_ directly interacts with the chemical or catalytic reaction system to reduce the Ea’, and another one portion of P_MW_ heats the reaction systems or catalyst beds to increase the temperature due to the MW thermal effect. Matter in the chemical reaction system or catalyst that absorbs MWs gives rise to two changes. First, the interior energy level of the molecules changes. In addition, the system or catalyst bed temperature increases. Therefore, when MWs irradiate chemical reaction systems or catalyst, the interior energy level of the molecules changes, which leads to a decrease in the Ea’, and the chemical reaction systems reaches a state of activation that leads to the chemical reactions occurring easily. When a suitable MW catalyst is used, the catalytic reaction via MW catalysis occurs easily. MWs are electromagnetic waves. An electromagnetic field must exist on the chemical reaction system or catalyst bed under MW irradiation, and microscopic particles in the chemical reaction systems including molecules, atoms and free electrons must/should obey the law of wave mechanics. An increase or change in the pre-exponential factor (See Table S7) indicates that the effective collision rate of molecules in the chemical reaction systems increases. In addition, an increase in the pre-exponential factor also indicates that the movement of these microscope particles in the chemical reaction systems change from disordered motion to ordered motion, which is govern by the law of wave mechanics. This change from disordered to ordered motion due to the MW electromagnetic field also indicates that MW irradiation can increase the energy efficiency of the system. At a constant MW frequency, a change in the pre-exponential factor depends on the MW electromagnetic field and P_MW_ (i.e., A∝ E P_MW_), which is complex and nonlinear under MW irradiation energy and MW electromagnetic field due to the oscillating electric field.

MW irradiation energy as well as the MW electromagnetic field cause a change in the molecular rotational energy level under MW irradiation, which leads to a decrease in the Ea’ for reactions involving a polar transition state, where the polarity is increased on going from the ground state to the transition state. At the same time, this effect will result in a change in the electron cloud distribution and/or bond length and/or bond angle. Therefore, this effect causes molecules in the chemical reaction systems to become activated, resulting in facile reactions.

MW irradiation enhanced the activation degree, which resulted in the activation of the molecules in chemical reaction systems and on the surface of catalysts. Interactions between the microwave electromagnetic waves and the molecules are not the same. Therefore, the activation degrees are different, especially for polar and non-polar molecules. This difference resulted in the MW selective effect^13^,^28^ (See Fig. S5).

Explanations of the experiment phenomenon and reaction results under MW irradiation are shown in S section II. Further study of other reaction systems and their interaction rules as well as the matching relationship of the interactions between microwave electromagnetic waves and molecules was investigated. Different chemical reactions should correspond to different suitable MW catalysts.

In summary, we have elucidated the nature of the microwave-driving force for speeding up chemical reactions. The model of interactions between the MW electromagnetic waves and molecules indicates that MW irradiation is partially transformed to reduce the Ea’ and exhibited a MW catalytic effect. The nature and mechanism of the MW irradiation on the chemical reaction was elucidated. An explanation was provided for the experimental phenomena and chemical reaction results from MW irradiation of chemical reactions. Our findings challenge the classical view of MW irradiation as a heating method and the controversial “MW non-thermal effect”. Our findings open a promising avenue for the development of new MW catalytic reaction technologies and MW catalysts.

## Additional Information

**How to cite this article**: Zhou, J. *et al.* A new type of power energy for accelerating chemical reactions: the nature of a microwave-driving force for accelerating chemical reactions. *Sci. Rep.* **6**, 25149; doi: 10.1038/srep25149 (2016).

## Supplementary Material

Supplementary Information

## Figures and Tables

**Figure 1:**
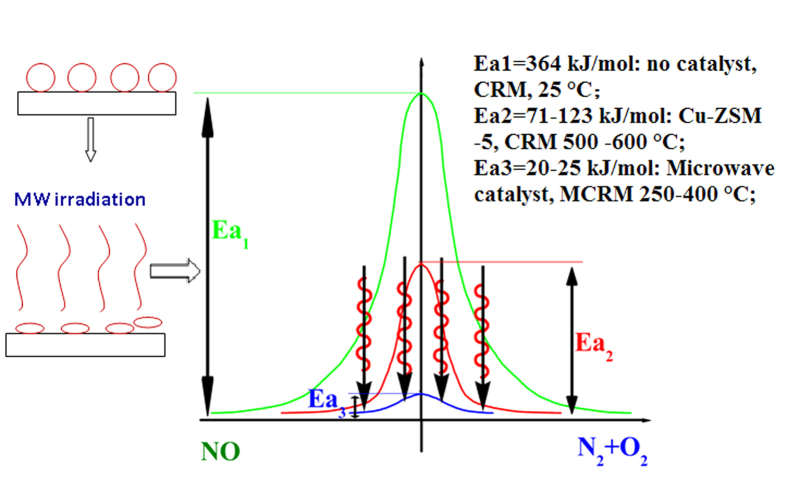
MW irradiation is a new type of power energy for speeding up chemical reactions.

**Table 1:** Comparison of the reaction temperature of the catalytic decomposition of NO or NO reduction in the MCRM and CRM.

Reaction/Conditions	Catalysts	NO conversion (%)	Reaction temperature/ Residence time
CRM, NO decomposition	Cu-ZSM-5	maximum, 53.6	550 °C, τ = 1.2 s
CRM, NO decomposition	Cu-ZSM-5	maximum, 65	550 °C, τ = 2.25 s
CRM, NO decomposition	Cu-ZSM-5 + CuO	62.1	550 °C, τ = 2.25 s
MCRM, NO decomposition	Cu-ZSM-5	56.6	250 °C,τ = 1.25 s
MCRM, NO decomposition	Cu-ZSM-5 + CuO	98.6	350 °C,τ = 2.25 s
MCRM, NO decomposition	BaMnO_3_	93.7	250 °C, W/F = 1 g s cm^−3^
CRM, NO decomposition	BaMnO_3_	45.4	650 °C, W/F = 1 g s cm^−3^
MCRM, NO reduction with AC	No catalyst	92.5	400 °C
CRM, NO reduction with AC	No catalyst	92.8	600 °C

**Table 2:** Apparent activation energies (Ea’) for the direct decomposition of NO.

Mode/Temperature conditions	Catalyst	Ea’ (kJ/mol)^a^	References
Direct decomposition of NO	–	364	40
CRM, 579–733 K	Cu/ZSM-5	123	41
CRM,500–650 °C	Cu-ZSM-5	71–121	42
CRM,500–650 °C	Cu-ZSM-5	75.6	this work
MCRM,200–250 °C	Cu-ZSM-5	25.04	this work
MCRM,120–200 °C	CuO-Cu-ZSM-5	22.47–25.73	this work
MCRM, 360–400 °C	CuO-Cu-ZSM-5	19.55–22.81	this work

^a^Ea’ was calculated using the Arrhenius equation; 
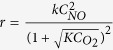
 was used to calculate the kinetic data^41^.

**Table 3:** Apparent activation energies (Ea’) for the direct decomposition of NO.

Mode/Temperature conditions	Catalyst	Ea’ (kJ/mol)^b^	References
MCRM, 200–250 °C	BaMnO_3_	33.4	this work
MCRM, 200–250 °C	BaFeO_3_	46.7	this work
CRM, 600–650 °C	BaMnO_3_	194.5	this work
CRM, 600–650 °C	BaFeO_3_	197.5	this work

^b^Ea’ was calculated using the Arrhenius equation; First-order kinetics with respect to NO were used to calculate the kinetic data^43^.
