# Supplementary material for: A new type of power energy for accelerating chemical reactions: the nature of a microwave-driving force for accelerating chemical reactions
Source: Sci Rep. 2016 Apr 27;6:25149. doi: 10.1038/srep25149 (PMC4846869; doi:10.1038/srep25149)
Supplement: Supplementary Information [file srep25149-s1.pdf]

Supplementary Information

A new type of power energy for accelerating chemical reactions: the nature of a microwave-driving force for accelerating chemical reactions

Jicheng Zhou*, Wentao Xu, Zhimin You, Zhe Wang, Yushang Luo, Lingfei Gao, Cheng Yin, Renjie Peng & Lixin Lan

Key Laboratory of Green Catalysis and Chemical Reaction Engineering of Hunan Province, School of Chemical Engineering, Xiangtan University, Xiangtan 411105, Hunan Province, China

***Corresponding author. Jicheng, Zhou; Tel.:+86-0731-58298173;**

E-mail address: zhoujicheng@sohu.com

S1. Experimental section

S1.1Preparation of the catalysts

S1.1.1 Preparation of the CuO-Cu-ZSM-5 catalyst

Cu-ZSM-5 was prepared using the ion-exchange method. H-ZSM-5 (Si/Al=33 supplied by the Changling Petroleum Refining Catalyst Plant, China) was exchanged with an aqueous solution of 0.01 mo1/L $Cu(AC)_2$ (AR grade, Shenyang Agent Company, China) according to a H-ZSM-5 to aqueous solution ratio of 15 g/L. Then, the solution was adjusted to a pH of 7 using ammonia followed by stirring for 12 h at 50 ℃. After filtration, the sample was washed with deionized water, dried at 100 ℃ for 10 h and calcined at 500 ℃

for 6 h. Ion exchange was performed 2 times to yield the final Cu-ZSM-5 zeolite catalyst containing 5 wt% of Cu.

The CuO-Cu-ZSM-5 catalyst was prepared using a mechanical mixing method. CuO was added to Cu-ZSM-5 and ground sufficiently and uniformly mixed in a mortar.

S1.1.2 Preparation of the $BaMnO_3$ and $BaFeO_3$ catalysts

The $BaBO_3$ (B=Mn, Fe) mixed oxides were prepared using the sol-gel method. $Ba(NO_3)_2$ (AR grade) and $Mn(NO_3)_2$ (AR grade) or $Fe(NO_3)_3$ (AR grade) were dissolved in deionized water according to a molar ratio of $BaBO_3$ (B=Mn, Fe). Citric acid and glycol were added to the aqueous solution, where the number of moles of the complexing species was 1.25 times that of the metal ions (i.e., Ba^{2+} and Mn^{2+} or Fe^{3+}). The aqueous solutions were stirred at 80 ℃ for 2.5 h to obtain spongy amorphous gels. The acquired gels were dried at 120 ℃ overnight and submitted to decomposition in air at 400 ℃ for 2 h followed by calcination at 700 ℃ for 4 h.

S1.1.3 Preparation of AC

Activated carbon (designated as AC, Φ3.0 mm, Sinopharm Chemical Reagent Co., Ltd.) was pretreated with an aqueous solutions consisting of HNO_3 (20 %) followed by drying at 80 ℃ for 12 h.

S1.1.4 Preparation of the $MeS/\gamma\text{-}Al_2O_3/BaMn_{0.2}Cu_{0.8}O_3$ (Me=Ni,Co) catalysts

The NiS and CoS nanoparticles were prepared by the solvothermal method. The $BaMn_{0.2}Cu_{0.8}O_3$ samples were prepared with sol-gel method, and the acquired gels were dried at 120 ^oC overnight and submitted to decomposition in air at 400 ^oC for 2h and then calcined at 700 ^oC for 4 h.

The MeS/γ-Al_2O_3/$BaMn_{0.2}Cu_{0.8}O_3$ (Me=Ni,Co) catalysts were prepared using a mechanical mixing method. MeS (Me=Co, Ni) and $BaMn_{0.2}Cu_{0.8}O_3$ (30 % by weight NiS and 40 % by weight $BaMn_{0.2}Cu_{0.8}O_3$, or 30 % by weight CoS and 60 % by weight $BaMn_{0.2}Cu_{0.8}O_3$, respectively) were added to γ-Al_2O_3 (surface area: 200 m^2/g), and the mixture was ground and mixed uniformly in a mortar. Then, the powders were formed into pellets by compression and sieved to 0.180-0.850 mm.

S1.2 Activity evaluation

S1.2.1 Microwave reactor system

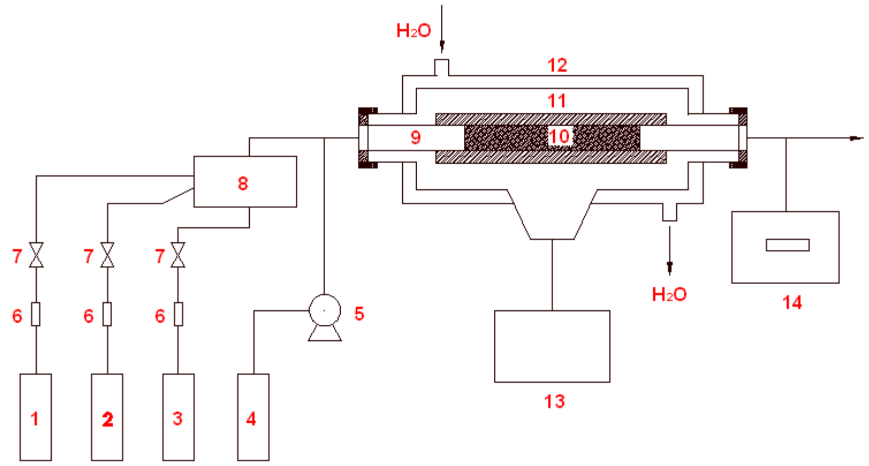

Figure S1 Schematic diagram of the microwave catalytic reactor system

1. NO; 2. O_2; 3. N_2; 4. Storage tank; 5. Metering pump; 6. Mass flow meter; 7. Valve; 8. Premixer; 9. Quartz reactor; 10. Fixed bed; 11. Thermal insulation; 12. Resonant cavity; 13. Microwave generator; 14. On-line NO_x analyser.

A multi-mode microwave catalytic reactor system was applied to investigate the role of microwave irradiation on a continuous flow of gas-solid catalytic reaction system. The experimental diagram is shown in Fig. S1. The reactor consisted of a microwave generator

system and a reaction system. The microwave energy was supplied by a 2.45 GHz microwave generator, and the power could be adjusted continuously in a range of 0-1000 W. The magnetron microwave source was connected through a rectangular waveguide to a resonant cavity (Φ28 mm*320 mm, the space of microwave irradiation is not only the catalyst but the whole cavity). A quartz tube (i.d.10 mm and 540 mm in length) at the centre of the cavity was designed to accommodate the experiments. The catalyst was placed in the middle of the quartz tube, and both ends of the tube were sealed with asbestos. Reliable monitoring of the reaction temperature is nontrivial but absolutely critical to the investigation of the microwave effects. Therefore, the temperature of the reaction bed was provided by microwave thermal effect and precisely measured by a modified thermocouple probe, which was coated with a tin-aluminium alloy to shield the microwave irradiation of the modified thermocouple itself and inserted into the catalyst bed. The advantages of this microwave reactor system are as follows:

(1) Continuously adjustable microwave power in the range of 0-1000 W with a frequency of 2450 MHz.

(2) The temperature of the catalyst bed can be precisely controlled by a microcomputer.

(3) The temperature of the catalyst bed can be measured precisely.

(4) The microwave source emits microwaves and irradiates the reaction tube, and the circulating water system is surrounds the periphery of the furnace chamber to absorb the microwaves to avoid damage to the magnetron caused by the reflected microwaves, which could protect the magnetron and allow the microwave catalytic reaction to continue to run for a long period of time.

S1.2.2 Activity tests

For the direct catalytic decomposition of NO over the CuO-Cu-ZSM-5 catalysts, the reactant gas was composed of NO (molar fraction, 0.1%), O_2 (molar fraction, 5.88%) and N_2 as the balance gas. All of the prepared catalysts had a granule size of 300-700 µm. The flow rate of the feed was 160 ml min^{-1} (GHSV = 1600 h^{-1}, τ = 2.25 s).

For the direct catalytic decomposition of NO over the $BaMnO_3$ and $BaFeO_3$ catalysts, the catalysts (2.0 g, 20-60 mesh) were used for each run, and the reactant feed rates were fixed at W/F = 1 g s cm^{-3}, where W and F are the catalyst weight and the total flow rate of reactant gas (F=120 ml min^{-1}), respectively.

For the reduction of NO by AC, the reactant gas was composed of NO (molar fraction, 0.1 %) and O_2 (molar fraction, 5.8 %) and N_2 as the balance gas. Ten millilitres of catalyst were used for each run, and the space velocity (GHSV) was 1020 h^{-1}.

For comparison, the CRM and MCRM were carried out in the same size reactor with the same reaction bed amount under identical conditions. In the CRM, the reaction test was carried out using a microreactor device (MRT-6123; Beijing Xin Hang Shield Petrochemical Technology Co., Ltd.). The concentrations of NO and NO_2 in the outlet gas were analysed by an online NO_x analyser (42C, Thermo Environmental Instruments Co., Ltd., U.S.). In addition, the analysis system consisted of GC (Agilent 7890A) with a thermal conductivity detector and a Porapak Q column for N_2O analysis. In the MCRM, N_2 was the desired product, NO_2 was the by-product, and N_2O was not detected. Therefore, the NO conversion and N_2 selectivity was calculated using the following formulas:

$$X_{NO} = \frac{C_0(NO) - C_1(NO)}{C_0(NO)} \times 100\% \quad , \quad \mathrm{S}_{NO} = \frac{C_0(NO) - C_1(NO) - C_1(NO_2)}{C_0(NO) - C_1(NO)} \times 100\%$$

(X_{NO} represents the NO conversion, S_{NO} represents the N_2 selectivity, $C_0(NO)$ represents the NO concentration prior to the reaction, $C_1(NO)$ represents the NO concentration after the reaction, and $C_1(NO_2)$ represents the NO_2 concentration after the reaction.)

For the direct catalytic decomposition of NO over the $BaMnO_3$ and $BaFeO_3$ catalysts, the reactor contains a flow of feed gas (15 vol% H_2S in N_2, 60 ml/min) that passes through the quartz tube, and the hydrogen content was analysed using gas chromatography (Agilent GC 7890 A) with a thermal conductivity detector (TCD) and a Porapak Q column (80-100 mesh, with 6ft*1/8”*2.0 mm). The operation temperature of the column was 80℃. A sample of the MeS/γ-Al_2O_3/$BaMn_{0.2}Cu_{0.8}O_3$ catalyst (m=2 g) was transferred to the microwave catalytic reactor system. The system was initially purged with nitrogen gas to prevent air oxidation or moisture uptake. A continuous stream of N_2, was employed, and when the catalyst bed reached the required reaction temperature under microwave irradiation, the H_2S valve was opened. The exit gas was analysed by GC at four minute intervals until consistent results were obtained for each reaction condition. During H_2S decomposition, only H_2 and sulfur were produced without any other by-products. Therefore, the H_2 yield directly reflects the efficiency of H_2S decomposition, and the selectivity to hydrogen was always 100%. In addition, the H_2S conversion can be expressed as follows:

$$X_{H_2S} = \frac{Y_{H_2}}{V^o_{H_2S}} 100\%$$

where X_{H_2S} is the H_2S conversion based on the formation of hydrogen. $V^o_{H_2S}$ is the initial volume fraction of hydrogen sulfide (15 % here).

S2. Results and discussion

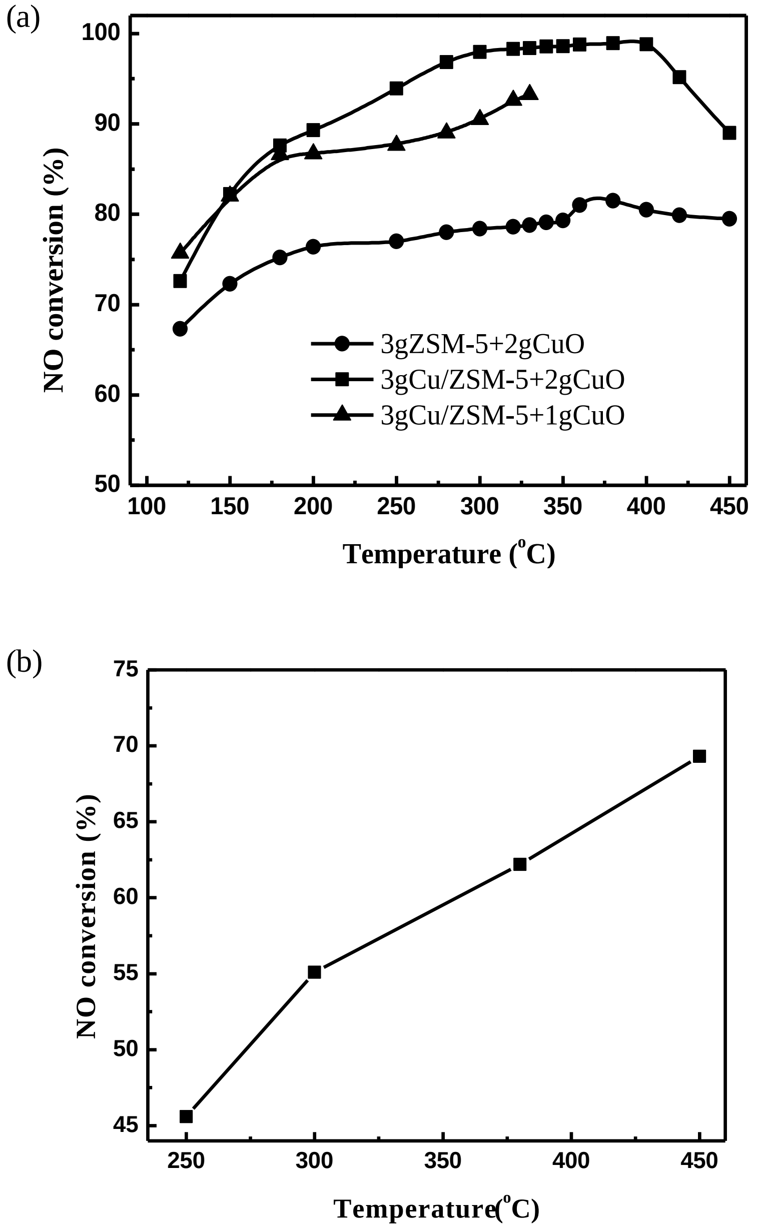

Figure S2. Effect of temperature on the direct decomposition of NO over 3 g Cu ZSM-5+2 g CuO (■), 3 g ZSM-5+2 g CuO (●), 3 g Cu ZSM-5+1 g CuO (▲) (a) and single 4 g CuO (b) in the MCRM. (Gas mixture: 1000 ppm NO, 5.88% O_2, N_2 as a balancing gas, GHSV = 1600 h^{-1}).

(a)

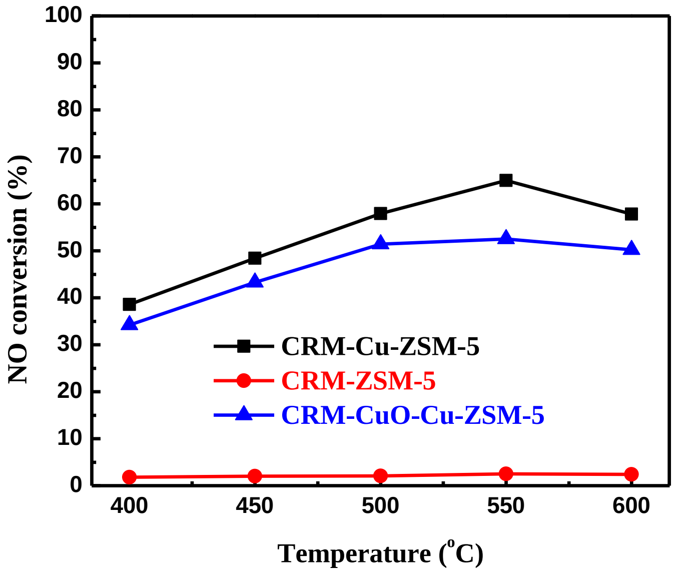

(b)

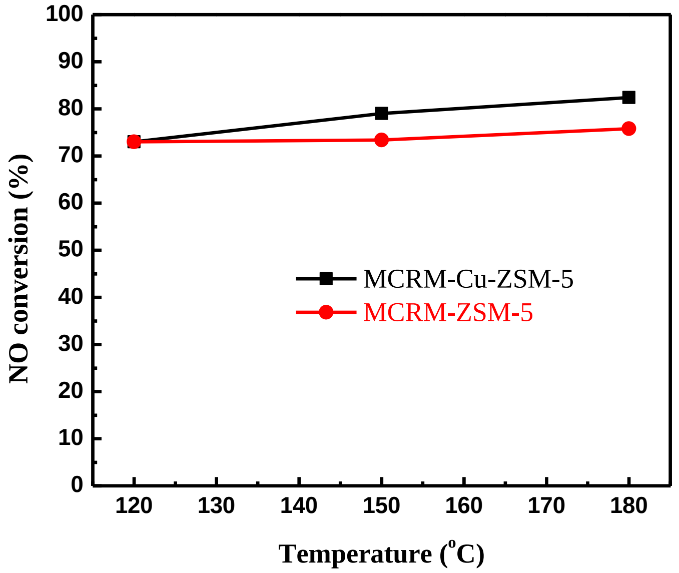

Figure S3. (a). Activity of NO conversion over 4 g ZSM-5, 4 g Cu-ZSM-5 and 3 g Cu-ZSM-5+1 g CuO in the CRM. (Gas mixture: 1000 ppm NO, 5.88% O_2, N_2 as a balancing gas, GHSV = 1600 h^{-1}).

(b). Activity of NO conversion over 4 g ZSM-5 and 4 g Cu-ZSM-5 in the MCRM. (Gas mixture: 1000 ppm NO, 5.88% O_2, N_2 as a balancing gas, GHSV = 1600 h^{-1})

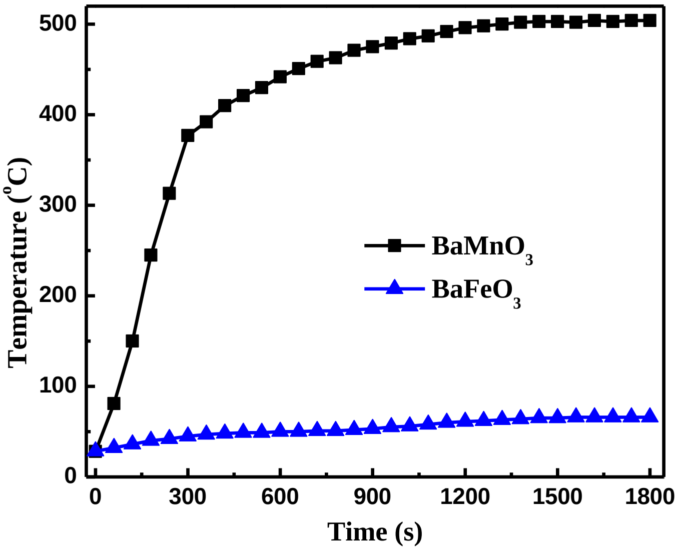

Figure S4. Microwave heating behaviour of the $BaMnO_3$ and $BaFeO_3$ catalysts in the MCRM using a microwave power of 150 W.

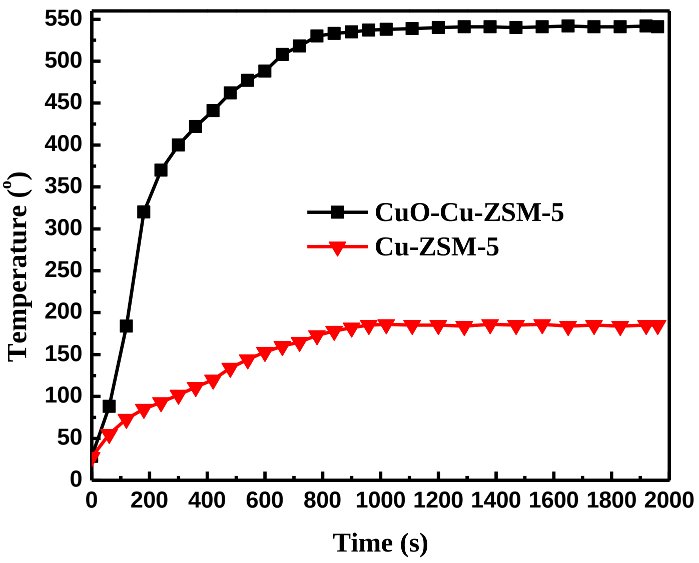

Figure S5. Microwave heating behaviour of CuO-Cu-ZSM-5 and single Cu-ZSM-5 in the MCRM using a microwave power of 150 W.

As shown in Fig. S5, the stable temperature of the bed was maintained at only 185 ℃ for single Cu-ZSM-5 under microwave irradiation for approximately 30 min at a microwave power of 300 W. Comparatively, the stable temperature of the bed was maintained at 541 ℃ for CuO-Cu-ZSM-5 at the same microwave power and irradiation time. The results indicate that MW adsorption property of the CuO-Cu-ZSM-5 catalyst is stronger than that of the single Cu-ZSM-5 catalyst.

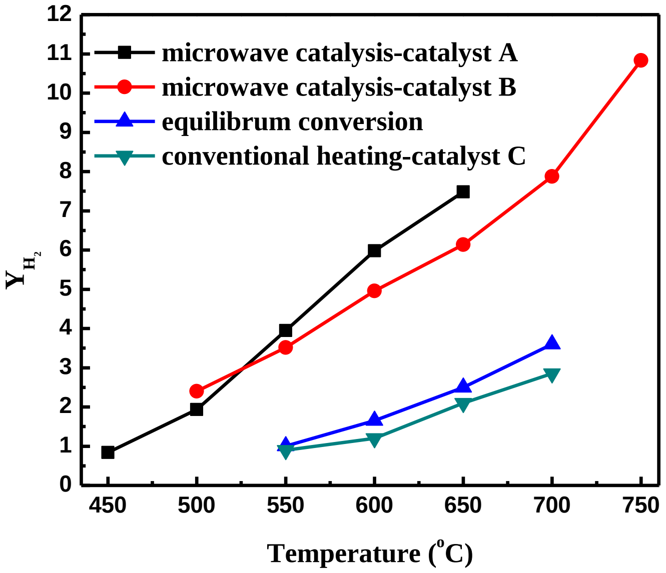

Figure S6. H_2 yield as a function of temperature with microwave catalysis-catalyst A: NiS/γ-Al_2O_3/$BaMn_{0.2}Cu_{0.8}O_3$; microwave catalysis-catalyst B: CoS/γ-Al_2O_3/$BaMn_{0.2}Cu_{0.8}O_3$; and conventional heating-catalyst C: MoS_2/γ-Al_2O_3.

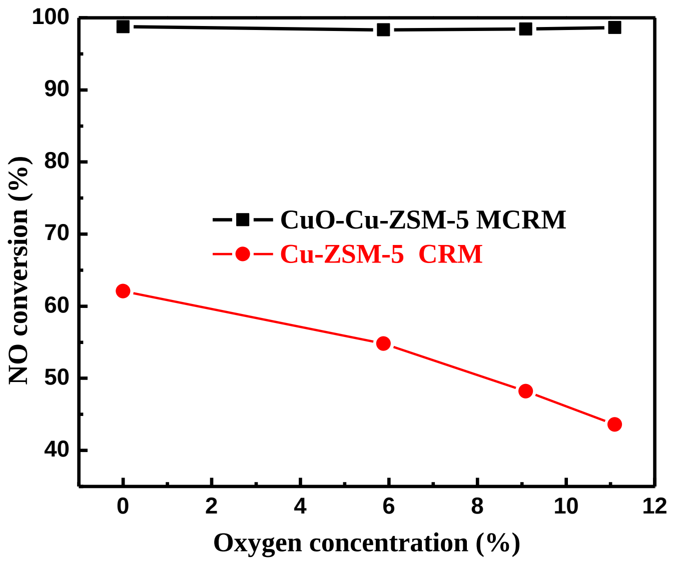

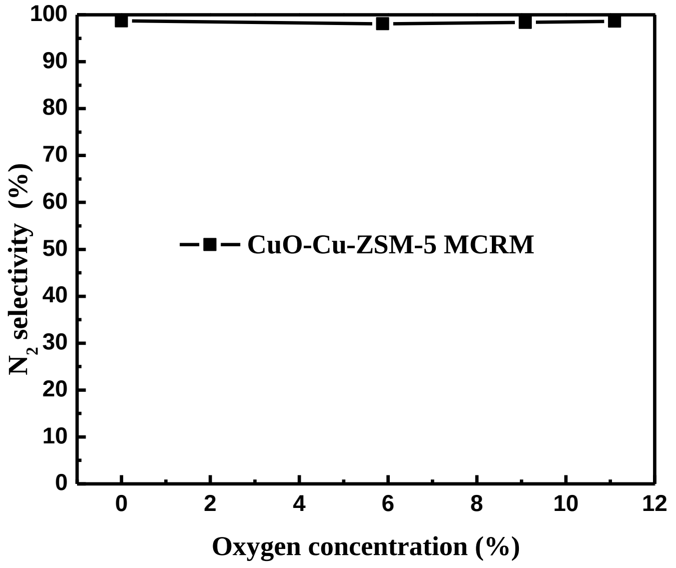

Figure S7. Effects of oxygen concentrations on NO conversion over CuO-Cu-ZSM-5 in the MCRM and Cu-ZSM-5 in the CRM (a). Effects of O_2 concentrations on the N_2 selectivity over CuO-Cu-ZSM-5 in the MCRM (b) (Reaction conditions: reaction temperature, 300 ℃ in the MCRM and 550℃ in the CRM, respectively; molar fraction of NO, 0.1%; N_2 as a balancing gas; and ambient temperature, 32˚C).

As shown in Fig. S7, the oxygen concentration does not influence the NO conversion or N_2 selectivity over the CuO-Cu-ZSM-5 MW catalyst in the MCRM. However, the N_2 conversion in the CRM decreased from 62.1% to 43.6% when the oxygen concentration

gradually increased from 0 % to 11.1 %. The CuO-Cu-ZSM-5 catalyst exhibited good stability to excessive oxygen in the MCRM even though Cu-ZSM-5 can be deactivated due to the adsorption of coexisting oxygen in the CRM. Moreover, the N_2 selectivity in the MCRM was more than 98.1 % (Fig. S7b). The high selectivity indicates that the undesirable consecutive reactions of NO with O_2 to generate NO_2 and NO conversion to N_2O barely occurred in the MCRM, and nearly the entire NO was directly converted to N_2. At the same time, we previously also found the NO conversion and N_2 selectivity were up to 99.8% and 99.9%, respectively for $BaMn_{0.9}Mg_{0.1}O_3$ catalyst even with the coexistence of 10% oxygen and low temperature of 250 °C.[S1]

The oxygen concentration does not influence the catalytic performance of the CuO-Cu-ZSM-5 MW catalyst for NO decomposition in the MCRM. This phenomenon is in agreement with our previous results.[S1,S2] The microwave selective effect under microwave irradiation may account for this phenomenon. Polar molecules are most easily activated by a microwave electromagnetic field. As we previously reported[S1,S2], the NO molecules that adsorbed on the active sites rather than O_2 molecules in the gas phase can be activated effectively by the microwave electromagnetic field. Although both O_2 and NO molecules can be physically adsorbed on the active sites, only physically adsorbed NO molecules in the MCRM become chemically adsorbed and further converted to N_2 and O_2. Simultaneously, O_2 molecules cannot be activated at this low reaction temperature due to the microwave selective effect, and they cannot react with NO molecules to form NO_2. Therefore, the CuO-Cu-ZSM-5 MW catalyst in the MCRM exhibited outstanding stability to excess oxygen.

Table S1 Apparent activation energies (Ea') of NO reduction by AC.

Mode/Temperature conditions	Ea' (kJ/mol)
CRM, 250-300 ℃	29.853
MCRM, 250-300 ℃	19.718

Table S2 Apparent activation energies (Ea') of direct catalytic decomposition of H_2S.

Mode/Temperature conditions	Catalysts	Ea' (kJ/mol)[a]	Reference
MCRM, 450-650 ℃	NiS/γ-Al_2O_3/$BaMn_{0.2}Cu_{0.8}O_3$	26.83	This work
MCRM, 650-750 ℃	CoS/γ-Al_2O_3/$BaMn_{0.2}Cu_{0.8}O_3$	19.46	This work
CRM, 650-850 ℃	NiS,MoS_2	69.04	S4
CRM, 650-850 ℃	CoS,MoS_2	59.21	S4
CRM, 500-900 ℃	γ-Al_2O_3	72	S5
CRM, 600-800 ℃	No catalyst	495.62	S6

[a] Ea' were calculated using the Arrhenius equation; Kinetic data was calculated according to ref.[S3].

Table S3 Effect of microwave input power on the balanced bed temperature over the CuO-Cu-ZSM-5 and single Cu-ZSM-5 catalysts.

Microwave power (W)	CuO-Cu-ZSM-5	Cu-ZSM-5
	Balanced bed T (℃)	Balanced bed T (℃)
400	299	196
600	367	225
800	412	242
1000	450	261

Table S4 Effect of the microwave input power on the balanced bed temperature over the $BaMnO_3$ and $BaFeO_3$ catalysts.

Microwave power (W)	$BaMnO_3$	$BaFeO_3$
	Balanced bed T (℃)	Balanced bed T (℃)
50	259	37
100	426	49
150	503	66

Table S5 Effect of the microwave input power on the balanced bed temperature over AC.

Microwave power (W)	Balanced bed T (˚C)
200	141
250	179
300	206
350	259
400	304

Table S6 Effect of the microwave input power on the balanced bed temperature over the MeS/γ-Al_2O_3/$BaMn_{0.2}Cu_{0.8}O_3$ (Me=Ni, Co) catalysts.

Microwave power (W)	NiS/γ-Al_2O_3/$BaMn_{0.2}Cu_{0.8}O_3$ Balanced bed T (˚C)	CoS/γ-Al_2O_3/$BaMn_{0.2}Cu_{0.8}O_3$ Balanced bed T (˚C)
400	520	682
600	581	730
800	624	788

Table S7 Effect of the reaction temperature range on the Ea' and pre-exponential factor (A) over the CuO-Cu-ZSM-5 catalyst.

reaction temperature range (˚C)	Ea' ($kJ.mol^{-1}$)	A (L.mol-1.h^{-1})
120-150	25.727	6.2336
150-180	22.468	47.284
180-200	14.790	119
200-250	25.378	8.167
250-280	54.542	0.01
280-300	59.652	0.0033
300-320	26.877	3.210
320-330	18.117	18.970
330-340	35.203	0.628
340-360	26.024	3.802
360-380	22.810	7.00
380-400	19.545	15.839

S section I

In fact, MW irradiation involves polarization, which is different from conventional heating and leads to the changes of in the internal energy level of molecules to achieve activation. One portion of the MW energy was adsorbed by molecules, and another portion of the MW energy was released by the MW thermal effect. Upon microwave irradiation of gas-solid phase or liquid-solid phase reactions in the presence of MW catalysts, the microwaves interact with molecules of the MW catalysts and simultaneously with molecules

that participate in the reactions. For other reaction systems, such as liquid-liquid phase reactions and solid-phase reactions, microwaves directly interact with molecules that participate in the reaction under MW irradiation.

The results indicate that the different MW adsorption properties of catalysts with the same catalytic active component exhibit different catalytic performances under MW irradiation. Therefore, K was dependent on the dielectric properties of materials or MW catalysts under MW irradiation.

$$\mathrm{Ea}_{\mathrm{MW}} = \frac{kE_{MW}P_{MW}}{1+kE_{MW}P_{MW}}E_{\mathrm{t}} \quad (9)$$

$\mathrm{Ea}_{\mathrm{MW}}$ can be calculated using Eq. (9). The decrease in the Ea' can be calculated using Eq. (10).

where Ea_0 is the activation energy barrier of the chemical reactions without MW irradiation. K is determined by the dielectric properties of the materials or MW catalysts as well as the MW frequency.

S section II

Explanation and elucidate about experiment phenomenon and reaction results under MW irradiation

*1 MW irradiation on chemical reactions can significantly reduce the Ea' to activate reactant molecules and simultaneously increase the pre-exponential factor (A), which leads to substantially speeded up chemical reaction rates that increase the reaction conversion or shorten the reaction time.

*2 MW irradiation of a chemical reaction can substantially reduce the Ea' to activate reactant molecules and simultaneously increase the pre-exponential factor (A). Therefore,

the reactions can be achieved under mild conditions compared to those of conventional heating. The reaction temperature decreased by as much as several hundreds of degrees centigrade under MW irradiation, and some reactions that do not proceed using conventional heating are successfully performed under MW irradiation. For the same reason, the reduction in the Ea’ of reactions may result in these reactions occurring under mild conditions. Therefore, the temperature of solid-phase chemical reaction systems is substantially lower under MW irradiation. For example, the temperature of solid calcination reactions or crystalline phase reactions was reduced by 100-300 ℃.

*3 MW irradiation interacts with matter or/and catalysts in chemical reaction systems, which resulted in a selective effect due to the difference in the interactions between the MW irradiation and matter. Therefore, the selective effect of MW irradiation resulted in an increase in the yield or change in the product distribution. This result is different from that obtained using conventional heating, where heat transfer does not produce a selective effect in the stationary state. For the same reason, the selective effect of MW irradiation results in an increase in the yield or change in the crystalline purity of solid-phase or liquid-solid phase reaction systems[S7].

*4 MW irradiation interacts with matter or/and catalysts in chemical reaction systems, which resulted in a selective effect. The selective effect of MW irradiation may cause the difference in speeding up forward and reverse reactions for reversible chemical reactions. Therefore, this result is very different from that of a catalyst using conventional heating that typically speeds up the forward and reverse reactions, which results in no selective effect. Therefore, the MW selective effect can increase the equilibrium conversion for a reversible

chemical reaction. For example, the equilibrium conversion of H_2 for the direct H_2S decomposition reaction increases from 5 % up to 11.2 % at same reaction temperature of 750 ℃ (Fig. S6) and from 6 % to 20 % at same reaction temperature of 800 ℃[S8]. Therefore, the MW selective effect disrupts the restriction of the reaction equilibrium conversion.

In our previous study[S7-S8], we reported that the MW selective effect for the microwave direct catalytic decomposition of NO resulted in no oxygen inhibition for competition reactions. Microwaves interact with polar molecules rather than with non-polar molecules, and microwave irradiation is beneficial for the activation of polar molecules. NO and O_2 molecules can be physically adsorbed on the active sites. However, only the physically adsorbed NO molecules became chemically adsorbed on the active sites and were further converted to N_2 in the MCRM. The physical adsorption of O_2 molecules is not enough to be activated at low temperatures under microwave irradiation, and the molecules do not react with NO to generate NO_2. These results indicated the elimination of oxygen inhibition for the direct decomposition of NO over the $BaMn_{1-x}Mg_xO_3$ catalysts in the MCRM[S7]. Therefore, microwave irradiation of the chemical reactions exhibited a microwave-selective effect.

S3. References:

S1 Xu, W., Zhou, J., Ou, Y., Luo, Y. & You, Z. Microwave selective effect: a new approach towards oxygen inhibition removal for highly-effective NO decomposition by Microwave catalysis over $BaMn_xMg_{1-x}O_3$ mixed oxides at low temperature under excess

oxygen. *Chem. Comm.* 51, 4073-4076 (2015).

S2 Xu, W., Zhou, J., You, Z., Luo, Y. & Ou, Y. Microwave Irradiation Coupled with Physically Mixed MeO (Me=Mn,Ni) and Cu-ZSM-5 Catalysts for the Direct Decomposition of Nitric Oxide under Excess Oxygen. *ChemCatChem* 7, 450-458 (2015).

S3 Reshetenko, T., Khairulin, S., Ismagilov, Z. & Kuznetsov, V. Study of the reaction of high-temperature H_2S decomposition on metal oxides (γ-Al_2O_3,α-Fe_2O_3,V_2O_5). *Int. J. Hydrogen Energy* 27, 387-394 (2002).

S4 Yumura, M. & Furimsky, E. Hydrogen sulphide adsorption and decomposition in the presence of manganese nodules. *Appl. Catalysis* 16, 157-167 (1985).

S5 Al-Shamma, L. & Naman, S. The production and separation of hydrogen and sulfur from thermal decomposition of hydrogen sulfide over vanadium oxide/sulfide catalysts. *Int. J. Hydrogen Energy* 15, 1-5 (1990).

S6 Kaloidas, V.E. & Papayannakos, N.G. Kinetic studies on the catatlyticdecompostion of hydrogen sulfide in a tubular reactor. *Ind. Eng. Chem. Res.* 30, 345-351 (1991).

S7 Panda, A.B., Glaspell, G. & El-Shall, M.S. Microwave synthesis of highly aligned ultra narrow semiconductor rods and wires. *J. Am. Chem. Soc.* 128, 2790-2791 (2006).

S8 Zhang, X. & Hayward, D.O. Applications of microwave dielectric heating in environment-related heterogeneous gas-phase catalytic systems. *Inorg. Chim. Acta* 359, 3421-3433 (2006).
